# Clinical results of carotid artery stenting versus carotid endarterectomy

**DOI:** 10.17712/nsj.2016.4.20160079

**Published:** 2016-10

**Authors:** Tuba Akinci, Eda Derle, Seda Kibaroğlu, Ali Harman, Feride Kural, Pınar Cınar, Munire Kilinc, Hakki T. Akay, Ufuk Can, Ulku S. Benli

**Affiliations:** *From the Department of Neurology (Akinci), Buyukcekmece Hospital, İstanbul, from the Department of Neurology (Derle, Kibaroglu, Kilinc, Can, Benli), the Department of Radiology (Harman, Kural), and the Department of Cardiovascular Surgery (Akay), Baskent University Faculty of Medicine, Teaching and Medical Research Center, Ankara, and from the Department of Neurology (Cinar), Batman Hospital, Batman, Turkey*

## Abstract

**Objective:**

To review our results of carotid artery stenting (CAS) and carotid endarterectomy (CEA).

**Methods:**

We evaluated the medical records of patients undergoing carotid artery revascularization procedure, between 2001 and 2013 in Baskent University Hospital, Ankara, Turkey. Carotid artery stenting or CEA procedures were performed in patients with asymptomatic carotid stenosis (≥70%) or symptomatic stenosis (≥50%). Demographic data, procedural details, and clinical outcomes were recorded. Primary outcome measures were in 30-day stroke/transient ischemic attacks (TIA)/amaurosis fugax or death. Secondary outcome measures were nerve injury, bleeding complications, length of stay in hospital, stroke, restenosis (ICA patency), and all-cause death during long-term follow-up.

**Results:**

One hundred ninety-four CEA and 115 CAS procedures were performed for symptomatic and/or asymptomatic carotid artery stenosis. There is no significant differences 30-day mortality and neurologic morbidity between CAS (13%) and CEA procedures (7.7%). Length of stay in hospital were significantly longer in CEA group (*p*=0.001). In the post-procedural follow up, only in symptomatic patients, restenosis rate was higher in the CEA group (*p*=.045). The other endpoints did not differ significantly.

**Conclusions:**

Endovascular stent treatment of carotid artery atherosclerotic disease is an alternative for vascular surgery, especially for patients that are high risk for standard CEA. The increasing experience, development of cerebral protection systems and new treatment protocols increases CAS feasibility.

Carotid artery stenosis being one of the leading causes of cerebrovascular diseases, is responsible for 20-25% of all strokes.^1^ The superiority of surgical therapy to medical therapy has already been proved in severe carotid artery stenosis (>70%) by large-scale studies on symptomatic and asymptomatic patients; including North American Symptomatic Carotid Endarterectomy Trial (NASCET),^2^ European Carotid Surgery Trial,^3^ and Asymptomatic Carotid Atherosclerosis Study.^4^ With the advent of endovascular techniques, carotid artery stenting (CAS) has emerged as an alternative to surgery for the treatment of severe carotid artery stenosis in 1990s.^5^ In randomized studies comparing 2 procedures; no significant difference was determined in terms of primary endpoints such as myocardial infarction (MI), stroke, death, or ipsilateral stroke in a 4-year period.^6^ After the Carotid Revascularization Endarterectomy versus Stenting Trial (CREST) study has shown that CAS is non-inferior to carotid endarterectomy (CEA) in terms of primary outcomes, the number of patients treated with endovascular therapy has increased.^6^-^8^ In this study, the short- and long-term clinical outcomes and restenosis rates of CAS and CEA procedures carried out at our center were compared in patients with asymptomatic and symptomatic carotid artery stenosis.

## Methods

This study included a total of 284 patients retrospectively, who underwent CEA or CAS for atherosclerotic stenosis in extracranial segments of carotid artery system in Baskent University hospital, Ankara from 2001 to 2013. Decision to proceed with CAS or CEA was based on the clinical evaluations performed by the departments of neurology, interventional radiology, and cardiovascular surgery and patient’s age, anatomic localization and severity of stenosis, symptom status, and comorbid conditions were taken into account. If the patient found to be eligible for both procedures, potential benefits and risks were explained in detail to patients and their relatives and the decisions as to which technique would be applied was made jointly with the patients and their relatives. As patient selection was retrospectively performed, no randomization between the 2 techniques could be performed. Either CEA or CAS performed angiographically for symptomatic internal carotid artery (ICA) stenosis of 50% and asymptomatic stenosis >70% were included, in this single center. Patients with totally stenosed ICA were excluded from CAS and CEA procedures.

Medical files of the patients obtained from the hospital archives was examined and the data including demographic properties such as age, gender; comorbid disorders including hypertension (HT), coronary artery disease (CAD), diabetes mellitus (DM), peripheral arterial disease (PAD), previous stroke, hyperlipidemia, and smoking; neurological symptom status related to carotid artery disease; severity of carotid artery stenosis; postoperative complications; new vascular events during follow-up; and restenosis of the target vessel was recorded.

The patients were categorized into 2 groups based on symptom status; patients with amaurosis fugax, stroke, transient ischemic attack (TIA), syncope and/or vertigo associated with carotid artery stenosis were considered as symptomatic, while patients without neurological symptoms were considered asymptomatic. Severity of carotid artery stenosis was determined by Carotid Doppler Ultrasonography (CDUS) according to Washington Criteria, Computed Tomography Angiography (CTA), Magnetic Resonance Angiography (MRA), and Digital Subtraction Angiography (DSA). Severity of carotid artery stenosis was measured by conventional angiography as previously described in NASCET study.^9^ A stenosis of 50% or greater was intervened in symptomatic cases and a stenosis of 70% or greater in asymptomatic cases.

Carotid artery stenting was performed by 2 interventional radiologists with procedural experience of more than 15 previous carotid stenting procedures. During these procedures, cerebral protection techniques employing filter systems (Spider TM, Ev3 Inc., Plymouth, MN, USA) were used in 111 cases; no embolic protection device was used in 4 cases due to the unavailability of those systems at the time of procedure. Carotid endarterectomy was performed under regional or local anesthesia by 2 separate cardiovascular surgeons with an operative experience of >15 previous endarterectomy procedures. Surgical procedures performed included primary repair, patch angioplasty, and eversion endarterectomy.

Data on major complications such as mortality, stroke/TIA/amaurosis fugax, and hyperperfusion syndrome and the minor complications such as local hematoma and nerve injury within 1-month period after the procedure were accessed from the medical records. The long-term rates of mortality, stroke/TIA/amaurosis fugax, and restenosis were reviewed. The medical data of patients attending regular control visits were obtained from patient records. Patients with no long-term follow-up after the procedure were called and invited to hospital for control visits. Patients attending control visits were questioned for complications, physically examined, and examined with CDUS for restenosis. All patients attending control visits gave written informed consent.

Study data were analyzed based on the definitions below: Mortality was defined as death from any cause; TIA was defined as a newly developed neurological deficit that recovers within 24 hours; stroke was defined as neurological deficit lasting for more than 24 hours and/or the presence of a lesion on the side of the procedure, with incresed diffusion on diffusion weighted sequences and decreased signal on apparent diffusion coefficient (ADC) sequences on MRI. Stroke, TIA, and amaurosis fugax on the same side as a procedure were defined as vascular complications.

First 30 days after the procedure were defined as the short-term and beyond 30 days as the ‘long-term’. A stenosis of more than 50% (symptomatic and asymptomatic) or total occlusion in CDUS was defined as restenosis. This study was approved by the Baskent University Institutional review Board and Ethics Committee (Project No: KA 13/197) and was supported by the Baskent University Research Fund.

### Statistical analysis

Statistical analyses were performed with IBM SPSS for Windows Version 21.0 (SPSS Inc., Chicago, IL, USA). Numeric variables were expressed as mean±standard deviation and median (min-max); categorical variables were expressed as number and percentage. Parametric test assumptions (normality of distribution and homogeneity of variances) were checked before statistical comparisons were made. Inter-group differences between numeric variables between 2 independent groups were tested with independent samples t test when parametric test assumptions were met and with Mann Whitney-U test otherwise. Categorical variables were compared between the groups using Chi-square test or Fisher’s exact test. A *p*-value of less than 0.05 was considered statistically significant.

## Results

The study included 176 patients undergoing carotid endarterectomy and 108 patients undergoing CAS, totaling 284 patients with a total of 309 intervened vessels (194 endarterectomy, 115 stent implantation). In the CEA group, 18 patients underwent bilateral intervention in separate sessions, while bilateral intervention was applied in separate sessions to 6 patients and in the same session to 1 patient in the CAS group.

The mean age of the CEA and CAS groups were 68.5±7.8 (49-86) years and 68.6±8.7 (41-84) years, respectively. Of 176 patients who underwent CEA, 138 (78.4%) were male; 76 (70.3%) of 108 patients who underwent CAS were male. The two groups were not significantly different with respect to age (*p*=0.914) and gender (*p*=0.178). The demographic and clinical properties of the patients were summarized on **Table 1**. Hyperlipidemia (*p*<0.001), peripheral artery disease (*p*=0.007), and smoking (*p*=0.002) were significantly more common in the CEA group than the CAS group. In addition, there were significantly more asymptomatic patients in the CEA group compared with the CAS group (*p*<0.001). In symptomatic patients, the time from the onset of symptoms to the procedure was less than 1 month in 59 (63.4%) patients in the CEA group and 65 (71.4%) in the CAS group (*p*=0.248).

**Table 1:** Clinical and demographic features of patients between CEA and CAS.

Clinical and demographic characteristics	CEA (n=194)	CAS (n=115)	*P*-value
	n (%)	
<70 years	103 (53)	59 (51.3)	
DiabetesMellitus	81 (41.8)	54 (47)	0.373
Hypertension	165 (85.1)	91 (79.1)	0.182
Coronary Artery Disease	122 (62.9)	60 (52.2)	0.064
Hyperlipidemia	134 (69.1)	51 (44.3)	<0.001
Smoking	84 (43.3)	38 (33)	0.002
Asymptomatic	94 (48.5)	20 (17.4)	<0.001
Symptomatic	100 (51.5)	95 (82.6)	<0.001
Stroke	55 (28.3)	55 (47.8)	
Transient Ischemic Attack	13 (6.7)	17 (14.8)	
Amaurosis fugax	10 (5.1)	10 (8.7)	
Vertigo	17 (8.8)	7 (6.1)	
Syncope	5 (2.6)	6 (5.2)	
*Stenosis*			0.983
50-70%	7 (3.6)	5 (4.3)	
≥70%	187 (96.4)	110 (95.7)	

CEA - carotid endarterectomy, CAS - carotid artery stenting

The analysis of CEA and CAS groups for short-term complications revealed no significant difference between the 2 (**Table 2**). Clinical presentation (asymptomatic vs symptomatic) and age (<70 years vs ≥70 years) did not significantly affect early vascular events (**Table 3**). Deaths in the CEA group occurred from heart failure in one patient and from stroke secondary to the occlusion of the target artery at the first postoperative day in the other.

**Table 2:** Comparison of the short-term complications between CEA and CAS.

Complications	CEA (n=194)	CAS (n=115)	*P*-value
	n (%)	
*Short-term complications*	24 (12.4)	21 (18.3)	0.211
Stroke/TIA/Amaurozis fugax	15 (7.7)	15 (13)	0.185
Local hematoma	9 (4.6)	2 (1.7)	-
Nerve injury	1 (0.5)	-	-
Exitus	2 (1)	-	-
Bradykardia/hypotension	1 (0.5)	2 (1.7)	-
Intracranial hematoma	-	2 (1.7)	
Myocardial infarction	-	-	-

CEA - carotid endarterectomy, CAS - carotid artery stenting

**Table 3:** The relation of vascular complication with clinical presentation and patient age.

Complications	Short-term stroke/TIA/amaurosis fugax		*p*-value
	CEA	CAS	
	n (%)		
Asymptomatic	4/94 (4.3)	1/20 (5)	1.000
Symptomatic	11/100 (11)	14/95 (14.7)	0.571
<70 years	7/103 (6.8)	5/59 (8.5)	0.759
≥70 years	8/91 (8.8)	10/56 (17.9)	0.171

n - no of complication/no of patients, CEA - carotid endarterectomy, CAS - carotid artery stenting

The mean duration of hospital stay was 5.9±5.8 days in the CEA group and 4.5±4.4 days in the CAS group. The shortness of hospital stay duration in CAS group was statistically significant (*p*<0.001). One hundred and thirty (67%) patients in the CEA group and 86 (74.8%) patients in the CAS group had long-term neurological examinations and CDUS tests. All other patients were invited to attend follow-up visits via telephone but some of them refused to do so while some others had died before. It was learned from patient relatives that 18 (10.3%) patients in the CEA group and 6 (5.5%) patients in the CAS group died during follow-up, but the exact cause of death could not be learned as the interviews were done via telephone. Death rate was not significantly different between the 2 groups (*p*=0.288).

In the CEA group with follow-up information, the mean follow-up duration was 24.4±23.7 (1-105) months while it was 17.1±23.4 (1-120) months in the CAS group (*p*=0.003). The analysis of the long-term complications of the patients with available follow-up data revealed that 4 (4.7%) in the CAS group and 9 (6.9%) in the CEA group developed ipsilateral stroke, TIA, and amaurosis fugax. No significant difference was observed between long-term complications in both groups (*p*=0.693). A subgroup analysis based on clinical presentation and age did not reveal any significant difference with regard to vascular complications (**Table 4**, **Table 5**).

**Table 4:** The relation of long-term vascular complication and restenosis rate with clinical presentation.

Complications	Asymptomatic		*p*-value	Symptomatic		*p*-value
	CEA(n=63)	CAS(n=18)		CEA(n=67)	CAS(n=68)	
Long-term vascular complication	3 (4.8)	2 (11.1)	0.307	6 (9)	2 (2.9)	0.165
Restenosis	8 (12.7)	3 (16.7)	0.701	11 (16.4)	3 (4.4)	0.045

CEA - carotid endarterectomy, CAS - carotid artery stenting

**Table 5:** The relation of long-term vascular complication and restenosis rate with age.

Complications	<70			≥70		
	CEA(n=70)	CAS(n=47)	*p*-value	CEA(n=60)	CAS(n=39)	*p*-value
Long-term vascular complication	4 (5.7%)	2 (4.3%)	1.000	5 (8.3%)	2 (5.1%)	0.701
Restenosis	14 (20%)	3 (6.4%)	0.075	5 (8.3%)	3 (7.7%)	1.000

CEA - carotid endarterectomy, CAS - carotid artery stenting

Nineteen (14.6%) patients in the CEA group and 6 (7%) patients in the CAS group developed target vessel restenosis. The restenosis rate was higher in the CEA group compared with the CAS group, although the difference was not statistically significant (*p*=0.133). There were non-significant correlations between restenosis rate and HT (*p*=0.3), HL (*p*=0.9), DM (*p*=0.5), CAD (*p*=0.4), and smoking (*p*=0.5). A subgroup analysis based on clinical presentation indicated a significantly higher restenosis rate in symptomatic patients undergoing CEA than those undergoing CAS (*p*=0.045) (**Table 4**). A subgroup analysis by age showed that restenosis rate was higher in patients younger than 70 years in the CEA group compared with the patients of the same age in the CAS group, although that difference did not reach statistical significance (**Table 5**).

## Discussion

Carotis artery stenting was first used for patients at high risk for CEA and has been shown that it may be efficiently and safely used for this group of patients.^10^ Randomized studies comparing CAS and CEA have reported similar short-term rates of stroke, MI, and mortality and long-term rate of ipsilateral stroke.^8^,^11^ CAS is now recommended as an alternative to CEA in severely symptomatic carotid artery stenosis and in selected asymptomatic patients.^12^

It has been suggested that operator’s experience, his/her medical branch, and patient selection criteria may influence complication rates of CEA and CAS.^13^-^15^ Therefore, success and complication rates may differ between centers.

In our study, the rates of 30-day stroke/TIA/amaurosis fugax were higher in the CAS group (13%) compared with the CEA group (7.7%), although the difference was not statistically significant. While there were 2 (1%) deaths within a 30-day period after the procedure in the CEA group, no death occurred in the CAS group. Previous studies have reported death or stroke rates of 6-9.6% for CAS and 3.2-6.3% for CEA.^16^-^19^ We suggest that the higher rate of short-term complications in our study may have resulted from differences in patient selection. Studies reported in the literature have not included the majority of patients with comorbid conditions and older than 75 years of age who are deemed high risk for surgical intervention.^9^ In some studies enrolling high-risk patients, the rates of short-term complications have been as high as in our study.^10^,^20^ When we analyzed patients having complications, we noticed that 8 of 16 patients with short-term complications were above the age of 75 years; furthermore, 50% of those who developed complication had a history of bypass surgery or comorbidities that create a thrombotic tendency such as chronic renal failure and atrial fibrillation. (Totally in CAS and CEA group 7 patients with chronic renal failure and 7 patients with atrial fibrillation). One additional cause may have been the differences in the definition and the time to emergence of complications in the other studies. We took into consideration all neurological complications that occurred in the first 30 days and either recovered or resulted in permanent disability. Many studies, however, have included complications causing permanent disability, while a few of them included all events lasting for more than 24 hours.^21^ While some studies have taken into account complications that lasted more than 24 hours after their onset, some others have taken complications lasting for 48-76 hours, and some others did so in complications lasting even beyond 1 week.^16^,^22^

There are only a few studies comparing endovascular treatment and surgical treatment in asymptomatic and symptomatic patients. ARCHER study found a 30-day stroke rate of 3.8% in asymptomatic cases and 10.9% in symptomatic ones.^10^ Our study, in compliance with the literature, found a higher rate of vascular complications within 30 days in symptomatic cases compared with asymptomatic ones in both the CAS and CEA groups.^10^

Carotid Stenting Trialists Colloboration (CSTC) meta-analysis comprising 3433 patients enrolled in EVA-3S, SPACE, and ICSS studies demonstrated that stroke and death were significantly higher in the CAS group than the CEA group in the first 120 days after the procedure, but the difference between the 2 procedures was related to the patient age at the time of procedure. According to that meta-analysis, rates of death and stroke within the first 120 days were the same in CAS 5.8% and CEA groups 5.7% in patients below the age of 70 years, but they were two-fold higher in the CAS group above the age of 70 years 12% for the CAS group versus 5.9% for the CEA group.^23^ Similarly, CREST study found that CAS was more beneficial with respect to primary endpoints at the periprocedural period including stroke, MI, and death for patients below the age of 70 years while CEA was more beneficial for those older than 70 years.^6^ Our study included a total of 147 cases above the age of 70. Short-term vascular complications were similar between CAS 6.8% and CEA 8.5% in those who were below the age of 70 years, while they were more frequent in the CAS group when the patients got older than 70 years, as reported in the literature. We did not find any difference between the group’s long-term complications with respect to the patient age at the time of procedure in either group.

Local hematoma and cranial nerve injury, which are among perioperative complications, have been reported more frequently with CEA than CAS,^11^,^24^ and our results were in compliance with literature results. Intra-procedural and post-procedural complications may lengthen duration of hospital stay with concomitant increase in treatment costs. In our study, duration of hospital stay was significantly shorter in the CAS group (*p*<0.001), as reported elsewhere.^11^ Although procedural complications are slightly more frequent at the periprocedural period with CAS, long-term rates of mortality and stroke were similar between CAS and CEA procedures.^6^,^25^-^27^ We also found that the long-term rates of stroke and death were similar in patients undergoing CAS or CEA, in compliance with the literature.

Restenosis in the target vessel may affect treatment success. In some studies, restenosis has been defined as stenosis of 50% or greater, and in some others, 70% or greater.^25^,^28^,^29^ Not every stenosis leads to new neurological problems; studies have reported restenosis rates varying between 6% and 14%, although only 1-5% of these cases have reported to suffer a new neurological event.^30^ In a randomized controlled multicenter study, the restenosis rates of CEA 6.3% and CAS 6% have been reported.^28^ In the same study, being symptomatic did not affect restenosis rate, while female gender, DM, and HL were independent predictors of restenosis.^28^ Another study found significantly greater restenosis rate in the endovascular treatment group compared with the surgery group.^25^ We found a higher, albeit non-significant, restenosis rate in CEA group than the CAS group. Unlike literature data, we found a significantly higher restenosis rate in symptomatic cases in the CEA group than the CAS group.

The limitations of our study were the retrospective, the low number of patients and the relatively short follow-up. Another limitation of our study was that it was performed at a single center.

In conclusion, the superiority of CEA and CAS over medical therapy has now been proved. Short- and long-term complications of both treatment modalities and long-term restenosis development affect procedural success. New studies are needed to increase procedural success rates.
